# Lugol Increases Lipolysis through Upregulation of PPAR-Gamma and Downregulation of C/EBP-Alpha in Mature 3T3-L1 Adipocytes

**DOI:** 10.1155/2020/2302795

**Published:** 2020-09-16

**Authors:** Cuellar-Rufino Sergio, Zepeda Rossana Citlali, Flores-Muñoz Mónica, Santiago-Roque Isela, Arroyo-Helguera Omar

**Affiliations:** ^1^Centro de Investigaciones Biomédicas, Universidad Veracruzana, Av. Luis Castelazo Ayala S/N, Col. Industrial Ánimas, Xalapa 91190, Veracruz, Mexico; ^2^Instituto de Ciencias de la Salud, Universidad Veracruzana, Av. Luis Castelazo Ayala S/N, Col. Industrial Ánimas, Xalapa 91190, Veracruz, Mexico; ^3^Facultad de Bioanálisis, Universidad Veracruzana, Odontólogos W/N, U. H. del Bosque, Xalapa 91190, Veracruz, Mexico; ^4^Biomedicine in Public Health Laboratory, Public Health Institute, Universidad Veracruzana, Av. Luís Castelazo Ayala S/N, Col. Industrial Animas, Xalapa 91190, Veracruz, Mexico

## Abstract

Overweight and obesity are defined as excessive and abnormal fat accumulation that is harmful to health. This study analyzes the effect of different concentrations of the lugol solution (molecular iodine dissolved in potassium iodide) on lipolysis in cultured 3T3-L1-differentiated adipocytes. The mature adipocytes were treated with doses from 1 to 100 *µ*m of lugol for 0.5, 6, and 24 h. The results showed that mature adipocytes exposed to lugol decrease their viability and increase caspase-3 activity with a lethal dose (LD50) of 473 *µ*m. In mature adipocytes, lugol decreased the total intracellular lipid content, being significant at doses of 10 and 100 *µ*m after 6 and 24 h of treatment (*P* < 0.01), and the accumulation of intracellular triglycerides decreased after 24 h of exposure to lugol (*P* < 0.05). Lugol treatment significantly increases the release of glycerol to the culture medium (*P* < 0.05). The levels of adipocyte-specific transcription factors C/EBP-*α* were downregulated and PPAR-*γ* upregulated after 30 min with lugol. These results indicate a lipolytic effect of lugol dependent on PPAR-*γ* and C/EBP-*α* expression in mature 3T3-L1 adipocytes.

## 1. Introduction

Obesity is a chronic disease that affects more than 650 million individuals worldwide and is characterized by excessive increase in adipose tissue due to an imbalance between caloric intake and energy expenditure [1]. Adipose tissue is the main storage site for energy reserves in the form of triglycerides although it also acts as an endocrine organ capable of regulating metabolism and homeostasis of fatty acids [2]. Lipolysis is a catabolic process employing stored triglycerides in adipose tissue for processing unesterified fatty acids and glycerol, in order to be used as energy substrates for the different organs and tissues. Therefore, excessive lipid accumulation generates adipose tissue dysfunction, which reduces lipolysis and increases lipotoxicity and the risk of developing other diseases such as diabetes mellitus II (DM-II) [3–5].

The 3T3-L1 cells have been widely used to study obesity and insulin resistance in adipocytes [6]. Differentiation of 3T3-L1 fibroblasts into adipocyte cells involves treatment with a cocktail of prodifferentiation agents such as insulin, glucocorticoids (dexamethasone), and isobutylmethylxanthine (IBMX), and once the cells are differentiated, they express characteristics of both white and brown adipose tissue, allowing the study of processes such as adipogenesis, lipid and glucose metabolism, and the action of hormones and drugs [6, 7].

Obesity is characterized by an increase in adipose tissue that results in the increase in adipocyte size and number [8]. The amount of adipose tissue is regulated by the inhibition of adipogenesis from preadipocytes to mature adipocytes [9] and lipolysis of adipose tissue [10]. Two key families of adipogenic transcription factors control the differentiation of fibroblast-like preadipocytes into mature adipocytes: CCAAT/enhancer-binding protein (C/EBP-*α*) and peroxisome proliferator-activated receptor-*γ* (PPAR-*γ*) [11, 12]. These transcription factors are ligand activated during differentiation and promote the expression of specific genes involved in the adipocyte phenotype and in glucose and lipid metabolism [11, 13–15].

Several reports have demonstrated the use of marine algae as potential therapeutic agents against obesity [16, 17], and among its components is iodine, a trace element that according to the species of algae can occupy between 0.06 mg and 624 mg/100 g dry weight [18]. Iodine *per se* has been reported to have antioxidant, oxidizing, apoptotic, and antiproliferative properties either in *in vivo* or *in vitro* models by a mechanism that involves the formation of iodolipids from polyunsaturated fatty acids such as arachidonic acid (AA) [19–22]. In 3T3-L1 preadipocytes and mature adipocytes, it is unknown whether they express the iodine transporters sodium/iodide symporter (NIS) although their expression in adipose tissue has been reported [23]. In the mammary and thyroid glands, iodine supplementation regulates the expression of PPAR-*γ* through iodinated lipids [19, 24, 25], inducing apoptosis [20, 21]. In iodine-deficient human trophoblasts, mRNA and protein levels of C/EBP-*β* and SERBF1 decrease [26], indicating that iodine could regulate lipid metabolism in adipose tissue. Therefore, the present study analyzes the effect of lugol on lipolysis, viability, and apoptosis in mature mouse 3T3-L1 adipocytes and the participation of the transcription factors PPAR-*γ* and C/EBP-*α*.

## 2. Methods

### 2.1. Cell Culture and Differentiation

3T3-L1 fibroblasts were initially maintained in Dulbecco's modified Eagle's medium (DMEM) supplemented with 8% fetal bovine serum (FBS), 2 mM/l glutamine, 100 U/l penicillin, and 100 *µ*g/ml streptomycin in a humidified atmosphere of 95% air and 5% CO_2_ at 37°C. To induce adipocyte differentiation, 3T3-L1 cells were allowed to grow to confluence and cultured with the differentiation medium containing 0.25 *µ*M/ml dexamethasone, 10 *µ*g/ml insulin, and 0.5 mM 1-methyl-3-isobutyl-xanthine. After 48 h exposure to the differentiation medium, cells were maintained for an additional 5-6 days in DMEM supplemented with 8% FBS and 5 *µ*g/ml insulin, and by day 8, about 90% of the 3T3-L1 cells differentiated in mature adipocytes showing typical visual. Mature adipocytes were then treated with 1, 5, 10, and 100 *µ*m of lugol (5 g molecular iodine dissolved in 10 g KI) for 0.5, 6, and 24 h in the serum-free media.

### 2.2. Determination of Total Lipid Accumulation by Oil Red O Staining

After treatment with lugol, cells were washed twice with phosphate-buffered saline (PBS) and fixed with 4% (v/v) paraformaldehyde for 1 h at room temperature. Thereafter, adipocyte cells were washed with isopropanol 60% (v/v) and allowed to dry. Subsequently, cells were stained with the Oil Red O solution (in 60% isopropanol) for 1 h and washed four times with water. Images of Oil Red O-stained cells were acquired and viewed using an inverted microscope (Motic Pro). Fat droplets stained red were extracted from cells using isopropanol, and the absorbance was measured at *λ* = 540 nm on a plate reader (Epoch microplate, Biotek, Winooski, VT, USA).

### 2.3. RNA Extraction and RT-PCR

NIS and PEN mRNAs were determined using RT-PCR. Total RNA was extracted using TRIreagent (SIGMA, St. Louis, MO, USA), and the cDNAs were synthesized from 5 *µ*g of RNA using M-MLV reverse transcriptase (Promega). The sequences of the sense and antisense primers used for amplification were as follows: glyceraldehyde-3-phosphate dehydrogenase, forward 5′ TGAAGGTCGGAGTCAACGG and reverse 5′ CCTGGAAGATGGTGATGGG and amplicon size 240 pb; NIS, forward 5′ TCAGTACATGCCACTGCTCG and reverse 5′ CAGTTACAGCTGCCATA and amplicon size 134 pb; PEN, forward 5′ GAACTCCGGAGCCCAAACAG and reverse 5′ GGCTCCGACCTGCCG and amplicon size 245pb. The amplification product was visualized in 2% agarose (Sigma) in 0.5 × TBE (0.045 M Tris-borate and 0.001 M EDTA) stained with ethidium bromide. The amplicon concentration was estimated and 500 ng was digested with 5–15 U of each restriction endonuclease *SmaI* for the NIS amplicon (two fragments 57 and 77 pb) and *SacII* for the PEN amplicon (two fragments 135 and 110 pb) at a volume of 50 *µ*l in the appropriate buffer, for 16 h. Visualization of the restriction fragments was done in 2–2.5% standard agarose gels.

### 2.4. Cell Proliferation Assay and Caspase-3 Activity

3T3-L1 preadipocytes (1 × 10^4^ cells/well) were incubated overnight in the DMEM medium with 10% FBS in 96-well plates. The 3T3-L1 cells were treated with lugol (0, 1, 10, 50, 100, 200, 400, 800, and 1000 *μ*m) for 0.5, 6, and 24 h. The growth was detected using the Thiazolyl Blue Tetrazolium Bromide (MTT) assay. MTT reagent was added to the 96-well plate and incubated for 3 h at 37°C. Then, the supernatant was gently eliminated, and 100 *μ*l of DMSO was added. The MTT-formazan product was measured by using a plate reader (Epoch microplate, Biotek, Winooski, VT, USA) at *λ* = 570 nm. Calculation of median inhibitory concentrations (IC_50_) was statistically analyzed by GraphPad Prism Version 6 software (GraphPad Software, 2016). Nonlinear regressions of log (inhibitor) vs response with four parameters were selected for IC_50_ estimation. The bottom and top parameters were constrained to 0% and 100%. Caspase-3 activity was assessed using Caspase-3 Colorimetric Assay Kit (BioVision Inc., Headquarters, Milpitas, CA, USA) following manufacturer's instructions.

### 2.5. Triglyceride and Glycerol Assay

After differentiation and treatment with lugol in 24-well plates, 3T3-L1 adipocytes were washed with PBS, scraped into 200 *μ*l PBS, and the suspension was centrifuged to 3500 rpm for 10 min. Intracellular triglycerides were quantified using a triglyceride kit according to the manufacturer's instructions (Kit triglycerides Quantification, Biovision K622). The results were expressed as % of triglycerides compared to the control. The amount of glycerol was measured by using a commercial Lipolysis Assay kit (Abcam ab185424). Cellular protein content was analyzed using a Bradford protein assay kit (Bio-Rad). Data were expressed as mean ± SD.

### 2.6. Western Blot Analysis

The cells are washed with ice-cold PBS 2 to 3 times, and RIPA lysis buffer (1% NP-40, 150 mM NaCl, 0.1% SDS, 50 mM Tris-HCl, pH 7.6, 10 mM EDTA, 0.5% deoxycholate, 1 mM PMSF, 1 mM sodium orthovanadate, 10 mM NaF, 10 mM *β*-glycerophosphate, 10 *μ*g/mL protease inhibitor, and phosphatase inhibitor cocktails) was added to extract cell proteins for 5 min at 4°C. Equal amounts of protein of each sample were separated by sodium dodecyl sulfate (SDS)-10% polyacrylamide gel electrophoresis (PAGE) and trans-blotted onto 0.2 *µ*m nitrocellulose membranes (Bio-Rad). Immunoblotting was performed with antibodies for C/EBP-*α* (SC-61, Santa Cruz Biotechnologies), PPAR-*γ* (D69), and *β*-actin (13E5) antibodies from Cell Signaling Biotechnologies at 1–1000 dilutions in 10% bovine serum albumin (BSA) in TBST overnight at 4°C. After three washes with TBST, secondary antibodies peroxidase horseradish were diluted (1 : 8000) in TBST containing 10% BSA and incubated for 1h at room temperature. The same blot was reprobed with *β*-actin antibody (Santa Cruz Biotechnologies) after stripping as an internal control. Signals were visualized with enhanced Amersham ECL Prime Western Blotting (Thermo Fisher Scientific Inc., México) according to the manufacturer's instructions. Images were acquired with ChemiDoc XRS + Imaging System Bio-Rad. Densitometry was made with ImageJ 1.5i software, and data are expressed as relative protein units.

### 2.7. Statistical Analysis

The data presented in all the figures are mean ± standard deviation (SD) of independent experiments (*n* = 3 to 5). In most cases, data from different experiments were normalized to the control value before being combined for statistical analyses. The data presented a normal distribution, and in all cases, parametric analyses were done. Differences among several groups were determined using one-way ANOVA followed by the Student–Newman–Keuls test using GraphPad Prism software (GraphPad Software Inc., San Diego, CA). *t-*test was used for comparison between two groups. *P* < 0.05 was considered significant.

## 3. Results

### 3.1. Cell Proliferation and Median Lethal Dose

**Figure 1:**
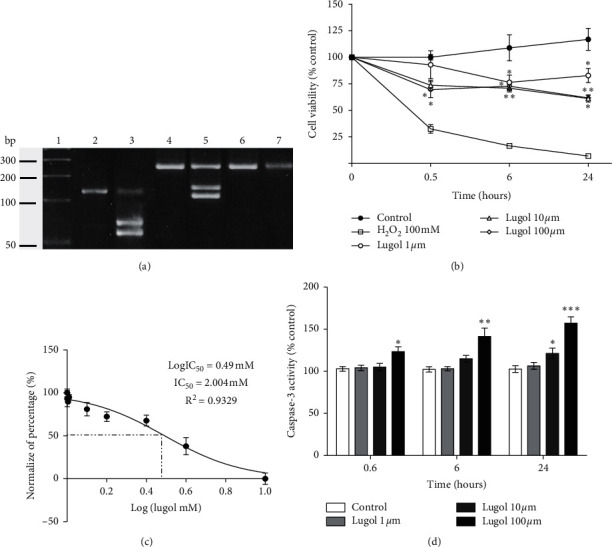
Expression of mRNA iodide transporters and inhibitory effects of lugol on mature 3T3-L1 adipocytes' growth. (a) Expression of NIS and PEN mRNAs on undifferentiated and differentiated 3T3-L1 (mature adipocytes). Representative electrophoresis (one of the three experiments with similar results is shown). DNA ladder (1), NIS amplicon (2), NIS plus SmaI (3), PEN (4), PEN plus SacII (5), and GAPDH as internal control (6-7). (b) Mature 3T3-L1 adipocytes were treated with lugol (0, 1, 10, and 100 *µ*M of lugol, *n* = 9). Cell proliferation was monitored using the MTT assay (see method) at 0.5, 6, 24 h following lugol treatment. OD *λ* = 570 value in mature 3T3-L1 adipocytes treated without lugol was defined as 100%. ^*∗*^*P* < 0.05; ^*∗∗*^*P* < 0.01; ^*∗∗∗*^*P* < 0.01 compared with the control group (vehicle). (c) Dose-dependent effects of lugol (0–1000 *μ*g/mL) for 24 h to determine the acute cytotoxicity dose (IC50). (d) After 0.6, 6, and 24 h, cellular caspase-3 activity was measured. Fold increase in activity was calculated based on activity measured in control (vehicle) cells. Each assay represents an independent experiment performed in triplicate. Data are presented as mean ± SD. n = 5. ^*∗*^*P* < 0.05, ^*∗∗*^*P* < 0.01, and ^*∗∗∗*^*P* < 0.01 vs vehicle cells.

Figure 1(a) shows the levels of mRNA of iodide transporters NIS and PEN in fibroblasts and mature 3T3-L1 adipocytes. The representative electrophoresis shows that mature adipocytes express both NIS and PEN mRNA iodine transporters; the amplicons' identity was determined by enzyme restriction digestion, and we got the expected products. To assess the effect of lugol on proliferation and apoptosis, 3T3-L1 preadipocytes and mature adipocytes were treated with 1 to 100 *µ*m lugol for 0.5, 6, and 24 h. Lugol decreases cell viability in a concentration- and time-dependent manner compared to control cells (Figure 1(b)). The lugol doses were increased from 1 to 1000 *µ*m to calculate the median lethal dose (LD_50_), and the results show an LD_50_ of 420 *µ*m (Figure 1(c)) at 24 h of treatment. Also, lugol increases caspase-3 activity at 10 and 100 *µ*m doses after 6 h of treatment (*P* < 0.05). In contrast, it is observed that lugol does not affect viability nor caspase-3 activity in control fibroblasts with doses of 1–100 *µ*m (Figure 1(d)).

### 3.2. Effect of Lugol on Lipid Droplet Formation and Triglyceride and Glycerol Content in Mature 3T3-L1 Adipocytes

**Figure 2:**
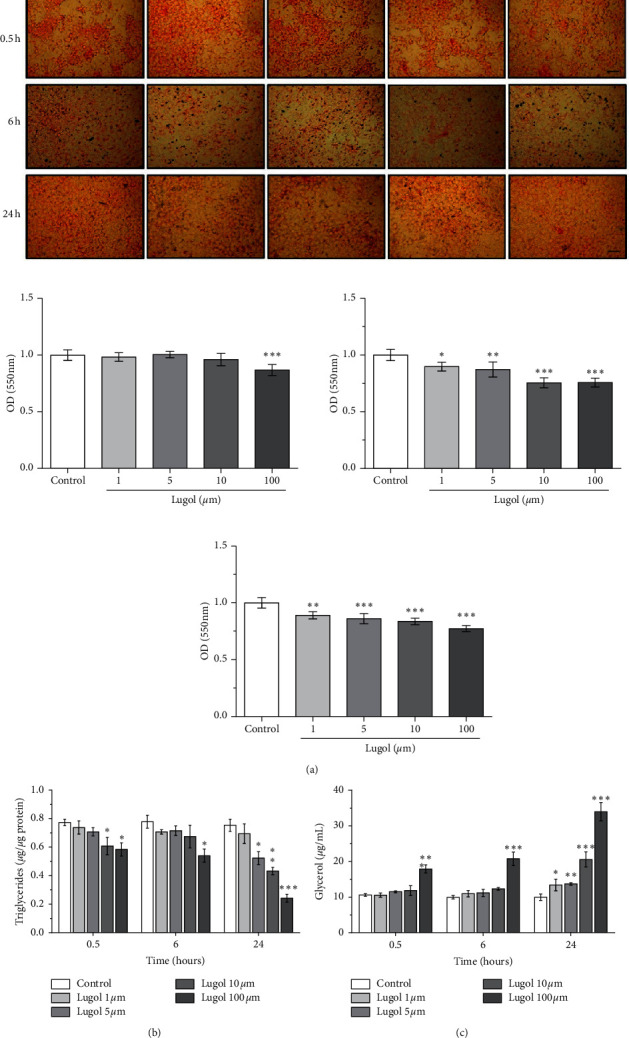
Effect of lugol on lipolysis in mature 3T3-L1 adipocytes. Mature 3T3-L1 adipocytes were treated in the serum-free medium with 1–100 *µ*M of lugol for 0.5, 6, and 24 h. (a) Micrographs of cells stained with Oil Red O and quantification; (b) triglyceride content and (c) lipolytic activity were measured from the amount of glycerol in the culture medium. Data are mean ± SD values from three experiments with triplicate determination. ^*∗*^*P* < 0.05, ^*∗∗*^*P* < 0.01, and ^*∗∗∗*^*P* < 0.001 vs control (vehicle) cells (*n* = 9). Scale bar is 100 *µ*m.

Differentiation of preadipocytes to mature adipocytes is associated with an increase in the number of adipocytes stained with Oil Red O by accumulation of total lipids. Lugol treatment inhibits lipid accumulation in adipocytes at 100 *µ*m for the first 30 min (*P* < 0.05). After 6 and 24 h, adipocytes treated with lugol significantly reduced lipid accumulation at concentrations of 1–100 *µ*m (*P* < 0.05) (Figure 2(a)). In Figure 2(b), it is shown that all concentrations of iodine (1–100 *µ*m) reduce intracellular accumulation of triglycerides compared with untreated adipocytes. Lugol at 10 and 100 *µ*m reduces triglyceride accumulation at 30 min of exposure by 30% at 10 *µ*m and 50% at 50 and 100 *µ*m (*P* < 0.01). However, at 24 h of treatment, the intracellular triglyceride content is reduced with doses from 5 µm of lugol (*P* < 0.001 vs control adipocytes). In Figure 2(c), an increase in the amount of glycerol released to the culture medium is observed in the adipocytes exposed to lugol although it was statistically significant with concentrations of 100 *µ*m at 30 min (*P* < 0.05) and at 6 h with 10 and 100 *µ*m (*P* < 0.05), while at 24 h of exposure with doses from 1 *µ*m, it was statistically significant (*P* < 0.001 vs control).

### 3.3. PPAR-y and C/EBP-*α* Expression in Mature 3T3-L1 Adipocytes Exposed to Lugol

**Figure 3:**
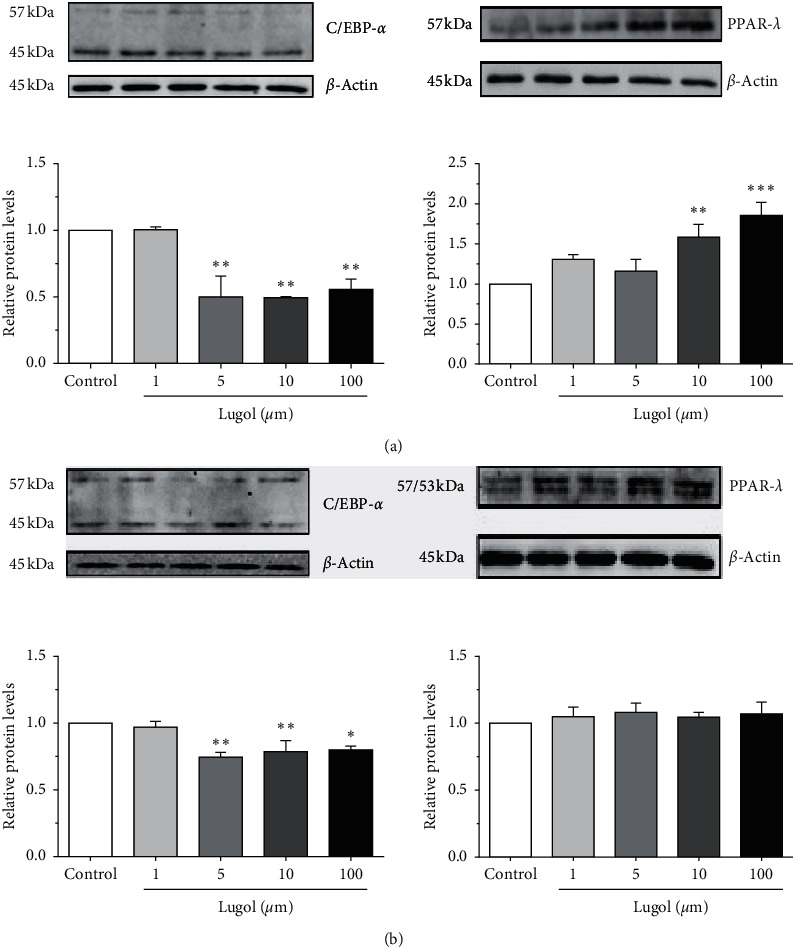
The effect of lugol on C/EBP-*α* and PPAR-*γ* protein expression in mature 3T3-L1 adipocytes. The cells were treated with 0, 1, 10, and 100 *µ*m of lugol for 30 min and 6 h and lysed in RIPA buffer for 1 h. The supernatant was subjected to SDS-PAGE. The blots were blocked and then were incubated with the primary and HRP-conjugated secondary antibodies. Detection was performed according to the enhanced chemiluminescence protocol. (a) 30 min after lugol treatment; (b) 6 h after lugol treatment. All quantitative data are mean ± SD (*n* = 3/group). ^*∗*^*P* < 0.05 and ^*∗∗*^*P* < 0.01 vs. control (vehicle) adipocytes.

Previous reports indicate that PPAR-*γ* is required for activation of the lipolysis regulatory network [27], and in transactivation assays, iodinated lipids have high affinity to the PPAR-*γ* transcription factor and can induce PPAR-*γ* gene expression [22]. When examining PPAR-*γ* expression in mature 3T3-L1 adipocytes treated with 1–100 *µ*m lugol, the results indicate a significant increase in PPAR-*γ* expression at doses of 10 *µ*m (*P* < 0.01) and 100 *µ*m (*P* < 0.001) at 30 min of treatment compared to control adipocytes (Figure 3(a)). However, lugol treatment after 6 h does not affect PPAR-*γ* expression in adipocytes (Figure 3(b)). On the other hand, C/EBP-*α* was downregulated from 5 *µ*m of lugol at 30 min and 6 h (Figure 3(b)).

## 4. Discussion

This study evaluated the ability of iodine to directly induce lipolysis in mature 3T31-L1 adipocytes. It has been previously reported that iodine uptake requires NIS and PEN transporters [28]. In this regard, previous studies have reported that adipose tissue expresses the iodine transporter NIS [23], which is in agreement with our results where we show that only mature adipocytes express the two mRNA of NIS and PEN transporters. This study shows that expression of NIS and PEN is regulated by transcription factors involved in adipocyte differentiation, such as the SREBP transcription factor [29, 30]. Transcriptional upregulation of the NIS gene by SREBP could be mediated by a functional binding site located in the NIS 5′-flanking region, which is not necessary for TSH-dependent NIS gene regulation. This indicates that regulation of the NIS gene by SREBP is important in extra thyroidal tissues that accumulate iodine and are not regulated by TSH [6].

Our results showed that exposing mature adipocytes to different concentrations of iodine induces lipolytic activity and accumulation of total lipids and triglycerides, and cell proliferation is reduced. These effects are accompanied by an increase in caspase-3 activity and glycerol release into the culture medium. These results agree with those reported in mice fed with an iodine-supplemented diet, where the decrease in cholesterol and triglycerides in the serum of mice fed with iodine-rich diet compared to unsupplemented mice was observed [31, 32].

It has been established that iodine can act as a free radical causing alterations in the cell membrane, and this explains the decrease in cell proliferation in our results. In accord, studies on cancer cell lines have revealed that iodine exerts antiproliferative and apoptotic effects through the BAX-caspase apoptotic pathway in breast cancer cell lines (MCF-7, MDA-MB-231, MDA-MB-453, ZR-75-1, and T-47D) with doses of 3 *µ*m of molecular iodine [21, 33], as well as apoptosis in DMBA-induced mammary gland tumors in the Wistar rat model [34, 35]. In our study, we also observe increases in caspase-3 activity at doses of 10 and 100 *µ*m, indicative of apoptosis. In contrast, low iodine doses have antioxidant effects and provide protection against oxidation although at high levels, it can induce oxidative damage and behave like a free radical [36–38]. This could be involved with damage to membranes, iodinated lipid formation, and cellular apoptosis.

The mechanism of action reported for iodine in other tissues involves the formation of iodolipids. These are ligands for the different PPAR isoforms, which by binding to specific DNA sequences induce the expression of genes related to metabolic processes, including the expression of PPAR-*γ* [3, 22]. In mature 3T3-L1 adipocytes exposed to rosiglitazone (PPAR-*γ* agonist), glycerol release is increased, through increased fatty acid oxidation and oxidative phosphorylation [39]. In addition, the activation of PPAR-*γ* by thiazolidinediones can decrease triglyceride content by increasing enzymes such as acetyl-CoA oxidase, enoyl CoA hydratase, acyl CoA dehydrogenase, and carnitine palmitoyltransferase [40–42]. Intracellular lipases (LPS) have been reported to have lipolytic function and are regulated by PPAR-*γ* through their promoter region, which contains elements responsive to PPAR-*γ* [43]. In our study, PPAR-*γ* increases its expression after 30 min treatment with lugol, and it is probable that the formation of iodinated lipids in the first hours of treatment into mature adipocytes might regulate the expression of the PPAR-*γ* gene, as it has been reported in mammary cancer cells [21]. On the other hand, C/EBP-*α* regulates the expression of PPAR-*γ* in adipocytes. Interestingly, our results contrast because lugol decreases C/EBP-*α* expression and increases PPAR-*γ* expression. A functional antagonism is possible between C/EBP-*α* and PPAR-*γ*, which favors PPAR-*γ* and causes downregulation of C/EBP-*α* [44]. Regarding the decrease in PPAR-*γ* and C/EBP-*α* after 6 h of treatment with lugol, it has been shown that the antiobesity effect for seaweeds involves a reduction in the expression of CEBP-*α* and PPAR-*γ* [45]. Moreover, CEBP-*α* and PPAR-*γ* proteins have a short life (<2.5 h) in adipocytes, and also ligand binding increases ubiquitin modification and proteasomal degradation [46, 47].

In conclusion, mature 3T3-L1 adipocytes express NIS and PEN mRNAs, which are involved in iodine uptake. Moreover, mature adipocytes exposed to lugol decreased the content of total lipids and triglycerides, and an increase in glycerol release was induced, also an induction of lipolysis and caspase-3 activation through regulation of PPAR-*α* and C/EBP-*α* was found. Thus, iodine nutrition alterations may be involved in the pathogenesis of obesity, suggesting that iodine is important on the maintenance of proliferation/lipolysis balance in adipose tissue. However, more research is required in order to know the exact mechanism of action of iodine in adipocytes.

## Data Availability

The datasets used and analyzed for this study are available from the corresponding author upon reasonable request.

## Additional Points

*Highlights*. (i) Mature adipocytes exposed to lugol reduce total lipids and triglycerides. (ii) Lugol induces lipolysis and increases glycerol release. (iii) Lugol regulated PPAR*γ* and C/EBP-*α* protein expression. (iv) Lugol inhibits adipocyte proliferation through caspase-3 activation.
